# Deep residual neural-network-based robot joint fault diagnosis method

**DOI:** 10.1038/s41598-022-22171-7

**Published:** 2022-10-13

**Authors:** Jinghui Pan, Lili Qu, Kaixiang Peng

**Affiliations:** 1 Institute of School of Automation, University of Science and Technology Beijing, Beijing, 100083 China; 2 Institute of School of Mechatronic Engineering and Automation, Foshan University, Foshan, 528231 China

**Keywords:** Engineering, Aerospace engineering, Electrical and electronic engineering

## Abstract

A data driven method-based robot joint fault diagnosis method using deep residual neural network (DRNN) is proposed, where Resnet-based fault diagnosis method is introduced. The proposed method mainly deals with kinds of fault types, such as gain error, offset error and malfunction for both sensors and actuators, respectively. First, a deep residual network fault diagnosis model is derived by stacking small convolution cores and increasing the core size. meanwhile, the gaussian white noise is injected into the fault data set to verify the noise immunity for the proposed deep residual network. Furthermore, a simulation is conducted, where different fault diagnosis methods including support vector machine (SVM), artificial neural network (ANN), convolutional neural network (CNN), long-term memory network (LTMN) and deep residual neural network (DRNN) are compared, and the simulation results show the accuracy of fault diagnosis for robot system using DRNN is higher, meanwhile, DRNN needs less model training time. Visualization analysis proved the feasibility and effectiveness of the proposed method for robot joint sensor and actuator fault diagnosis using DRNN method.

## Introduction

Recently, industrial robots have been widely used on many applications, such as automobile production line, aerospace, communication, and consumer electronics^1–3^. As an outstanding representatives of mechatronics technology, robot joint module integrates a large number of components, including hollow motor, servo driver, harmonic reducer, brake, encoder, into a limited space^4^. Considering complex and changeable working environment of robot joints, it is inevitable that kinds of faults will occur. If there is no fault diagnosis mechanism before fault happens, it will affect the production efficiency, product quality, and even expose human life in danger. Therefore, how to detect and locate the faults quickly and accurately is the most urgent thing^5^.

Researchers have been focused on Fault detection and fault tolerant control of robot joints for many years, and they have proposed a lot of practical fault diagnosis methods, including hardware redundancy and theoretical analysis-based fault diagnosis methods.

Among theoretical analysis-based robot joints fault diagnosis methods, observer is widely used^6,7^. Due to the fast convergence characteristic of sliding mode method, the error could attenuate as designed, therefore, it ensures the rapidity of observer, so it is used at all places in robot joint fault diagnosis^8,9^. In addition, backstepping algorithm, Takagi–Sugeno method as well as Luenberger observer are also applied for fault diagnosis^10–12^. However, most industrial robots are affected by disturbance or noise, so it is necessary to consider the effect of disturbance in robot fault diagnosis. For robot system, the first thing that comes to mind is to design disturbance observer. There are many methods for disturbance observer design, such as output feedback method^13^, nonlinear disturbance observer^14^ and feedback linearization disturbance observer design method^8^.

However, the trickiest problem of observer-based robot joint fault diagnosis method is that the gain of observer is very hard to design. At present, in the design of observer gain, the cost function should be determined first, and the observer gain is selected to minimize the cost function. The above design process extends the range for the value of the gain, which has a great impact on the performance of the observer. Stability of the observer is also an important consideration, and it is mostly guaranteed by Lyapunov function, which is very hard to find^15^.

The difficulty in gain design in observer-based robot fault diagnosis system promotes the research of robot fault diagnosis based on redundant sensors. Thanks to the development of sensors, sensors integrated with detection element, actuators and power supply have made a great breakthrough, such as magnetic detection chip, velocity measurement and gravity measuring elements^16^, and they have become very important feedback components in robot joint fault diagnosis system.

Three-dimensional gyroscope is used to measure the movement of the object. For a six degree of freedom robot joint system, low-cost MEMS magnetic, angular velocity and gravity sensors are used to estimate the joint angle of a rotating manipulator^17^. The sensor is installed at any position of the manipulator link in any attitude, and the attitude and position information of the joint system is obtained by above attitude measurement sensors.

However, the robot fault diagnosis system based on redundant sensors not only increases the structural complexity, but also increases the hardware cost of the system. In addition, additional sensors also increase the possibility of sensor failure in the system. With the development of artificial intelligence, the fault diagnosis method based on data-driven is developed. Robot system based on data-driven method only needs to be trained using fault data, which is easy to be collected when there is a fault on robot system, so there is no need to use redundant sensors.

Neural network (NN) method has the advantages of massively parallel, distributed processing, self-organization and self-learning capacity, so it is very popular on fault diagnosis^18–20^. The main stream of NN include basic perceptron, Feed-Forward NN (FFNN), long short-term memory (LSTM), CNN and RNN etc.^21–23^. Perceptron is the most original version of NN, which is just a simple neuron. As actual problems need more neurons to participate in the calculation, networks consist of many neurons are proposed, which are the NN in the ordinary sense. There are many variants of the NN. Zhang used multi-layer FFNN for mechanical fault diagnosis^24^. R. Sánchez researched Spur Gearbox with feed forward back propagation artificial NN to realize fault identification and classification^25^. Recently, Khouloud studied LSTM on reflective fiber fault detection, which reduced operation and maintenance expenses^26^. Albert also tried LSTM neural network for photovoltaic array fault diagnosis, and realized automatic feature extraction. Furthermore, to improve the feature extraction ability of neural network, convolution operation is adopted, where a convolution kernel is used to scan the full figure^27^. Chen researched CNN for bearing fault diagnosis^28^. Bo used adaptive label propagation technique combined with deep convolution variational autoencoder to achieve emerge fault diagnosis^29^. However, regular NN can’t extract adequate information needed by fault diagnosis architecture due to the simple structure of this network. FFNN requires abundant data sets to cover all situations, or it may fail. The matrix calculation of CNN is complex, especially in multi-layer NN structure. Moreover, CNN faces a severe problem, and that is the gradient loss in parameter updating by back propagation.

To overcome the gradient loss in parameter updating process, the RNN is used on fault diagnosis of robot actuator. The output of last layer is feed through to the next one, thus, the gradient loss problem which exists in deep learning NN is solved. A multi-scale cluster-graph convolution network with multi-channel RNN is proposed in^30^. Motor fault diagnosis under nonstationary conditions is studied in^31^. Also, the bearing fault location using RNN is researched in^32^. These scientific research on robot fault diagnosis using RNN extremely enriched robot application filed. Chen detailed the application of RNN on multiple domains^33^, and the bearing mechanic fault diagnosis under unknown situations is also a hotspot.

However, the existing NN fault diagnosis mostly focuses on the mechanical fault of robot actuators, without considering the fault classification of sensors and actuators at the same time. Both of the sensors and actuators of robot can have constant deviation and constant gain faults, and the sensors are seriously disturbed by noise. In addition, the difference of the fault data between the sensors and the actuator is very minor, and the use of conventional NN methods leads to low identification accuracy and long training and diagnosis time.

As an extension of our previous research in^34^,In this paper, a data-driven deep residual neural network fault diagnosis method is proposed for robot joints system. It covers many kinds of faults, including sensor and actuator faults, furthermore, the faults for both sensor and actuator are grouped in detail. Firstly, a deep residual neural network is constructed using small convolution kernel for fault diagnosis. Then, gaussian white noise obtained by MATLAB is injected into the fault data set to verify the noise immunity for the proposed deep residual network. Finally, the feasibility and effectiveness of the proposed deep residual network fault diagnosis method for robot joint sensor and actuator fault diagnosis are proved by simulation. The main contributions of this paper are as follows.

- (1) The robot sensor and actuator fault feedback data are very similar, which could be seen from our previous research [This is the article title of our previous research and it should be placed at reference], so this could result in ambiguous data feature boundary and this cause low diagnosis accuracy. The DRNN is adopted and the diagnosis architecture is well designed to eliminant gradient loss.
- (2) This paper gives a data fusion method for robot sensor and actuator feedback signals. Where the Gaussian white noise is considered to simulate the real working condition. The robot fault diagnosis using fused data simplifies dimension of input.
- (3) Comprehensive and comparison experiments are conducted, where the current popular NN methods are adopted to show their effectiveness and to compare with DRNN. The results show that DRNN owns faster converging speed and higher accuracy.

This paper is organized as follows: The model of robot joint is established in part II, and the fault data set is obtained according to the fault model in the first part. Then basic principle of DRNN is introduced in part III. In part IV, simulation and analytic visualization of fault classification results are conducted. The author summarizes the research at the end of this paper.

## Model and fault dataset

### Mathematical model of robot joint

The dynamic model of robot joint is established by Lagrange balance method from the point of view of energy^35^.

1 $$S_{0}{:}\;D(q)\ddot{q} + C(q,\dot{q})\dot{q} + G(q) = \tau ,$$

where $\tau$ is the vector for torque with dimension of n, $q,\dot{q},\ddot{q} \in R^{n}$ are state variables of angular position, angular velocity and angular acceleration, $D(q)$ and $C(q,\dot{q})$ are square matrix with dimension of n, denote inertia matrix and Coriolis force matrix respectively, $G(q) \in R^{n}$ is the gravity moment vector.

Let position and speed of joints be the state variables, and use variable substitution $x = [x_{1} \quad x_{2} ]^{T} = [q\quad \dot{q}]^{T}$, then (Eq. 1) could be rewritten as

2 $$S_{1} {:}\;\left\{ \begin{gathered} \dot{x} = Ax + Bf(q,\dot{q},\tau ) \hfill \\ y = Ex \hfill \\ \end{gathered} \right.,$$

where $x$ is the state variables for $S_{1}$,$y$ is the system output, $f(q,\dot{q},\tau )$ is the system coupling term, and the specific values of matrix $A$,$B$ and $E$ are as follows.

$$A = \left[ {\begin{array}{*{20}c} 0 & 0 \\ 0 & 1 \\ \end{array} } \right],B = \left[ {\begin{array}{*{20}c} 0 \\ 1 \\ \end{array} } \right],E = \left[ {\begin{array}{*{20}c} 1 & 0 \\ 0 & 1 \\ \end{array} } \right],$$

$$f(q,\dot{q},\tau ) = D^{ - 1} (q)[\tau - C(q,\dot{q})\dot{q} - G(q)].$$

### Fault model of robot joint actuator

Robot joint actuator faults can be roughly divided into two categories according to the mechanism on the occurrence of joint faults, and they are multiplicative fault and additive fault.

For $S_{1}$, when the actuator has multiplicative failure, the system (Eq. 2) could be written as follows

3 $$D(q)\ddot{q} + C(q,\dot{q})\dot{q} + G(q) = \rho \tau ,$$

where $\rho \in [0,1][0,1]$ is the effective factor of the actuator. A zero of $\rho$ means the actuator is completely broken while one means it works very well.

In addition to the effective factor $\rho$,there may be additional torque offset fault, and it could be represented in the following equation.

4 $$D(q)\ddot{q} + C(q,\dot{q})\dot{q} + G(q) = \tau + f_{a} ,$$

where $f_{a}$ is the actuator failure function, and its value is positively correlated with degree of damage.

Now both $\rho$ and $f_{a}$ would decide which kind of faults the actuator is currently suffering. The following four fault models can be established by further dividing the faults.

ErrA1: Constant deviation fault of actuator.

$$D(q)\ddot{q} + C(q,\dot{q})\dot{q} + G(q) = \tau + f_{a} ,(f_{a} \ne 0).$$

ErrA2: Constant gain fault of actuator.

$$D(q)\ddot{q} + C(q,\dot{q})\dot{q} + G(q) = \rho \tau ,(\rho \in (0,1)).$$

ErrA3: The actuator is completely stuck.

$$D(q)\ddot{q} + C(q,\dot{q})\dot{q} + G(q) = f_{a} ,(f_{a} \ne 0).$$

ErrA4: The actuator is completely broken.

$$D(q)\ddot{q} + C(q,\dot{q})\dot{q} + G(q) = 0.$$

Table 1 concludes above mentioned actuator fault types.

**Table 1:** Actuator fault type table.

$\rho$	$f_{a}$	Fault type
1	Not zero	Constant deviation fault
$0 < \rho < 1$	Zero	Constant gain fault
0	Not zero	Actuator stuck
0	Zero	Actuator broken

### Fault model of robot joint sensor

Similar to robot joint actuator fault, sensor fault can be divided into four groups, and they are constant deviation fault, constant gain fault and stuck fault as well as sensor completely broken. Sensors are used to detect system state, so sensor failure directly affects the state feedback of the system, which means the output of the system is directly affected.

Here, four types of sensor faults are listed and corresponding mathematical formula are also deduced.

ErrS1: Constant deviation fault of sensor.

$$\left\{ {\begin{array}{*{20}l} {\dot{x} = Ax + Bf(q,\dot{q},\tau )} \hfill \\ {y = Ex + f_{b} ,(f_{b} \ne 0)} \hfill \\ \end{array} } \right.,$$

where $f_{b}$ is the sensor failure function with the same dimension of $x$. $f_{b}$ is positively correlated with degree of damage.

ErrS2: Constant gain fault of sensor.

$$\left\{ {\begin{array}{*{20}c} {\dot{x} = Ax + Bf(q,\dot{q},\tau )} \\ {y = \lambda Ex,(\lambda \in (0,1))} \\ \end{array} } \right.,$$

where $\lambda$ is the effective factor of the sensor.

ErrS3: The sensor is at constant output point.

$$\left\{ {\begin{array}{*{20}l} {\dot{x} = Ax + Bf(q,\dot{q},\tau )} \hfill \\ {y = f_{b} ,(f_{b} \ne 0)} \hfill \\ \end{array} } \right..$$

When this happens, it means there is no reaction for the sensor when robot joint moves.

ErrS4: The sensor is at zero output point.

$$\left\{ {\begin{array}{*{20}l} {\dot{x} = Ax + Bf(q,\dot{q},\tau )} \hfill \\ {y = 0,(f_{b} \ne 0)} \hfill \\ \end{array} } \right..$$

The effective factor of the sensor is zero, and it means the sensor does not work anymore. The actuator and sensor faults are combined together, and considering (Eq. 1) and (Eq. 2), the expression after integration is given in (Eq. 5).

5 $$S_{1} {:}\,\left\{ {\begin{array}{*{20}l} \begin{gathered} \dot{x} = Ax + BD^{ - 1} (q)[\rho \tau { + }f_{a} - C(q,\dot{q})\dot{q} - G(q)] \hfill \\ \end{gathered} \hfill \\ {y = \lambda Ex + f_{b} } \hfill \\ \end{array} .} \right.$$

### Fault data acquisition

**Figure 1:**
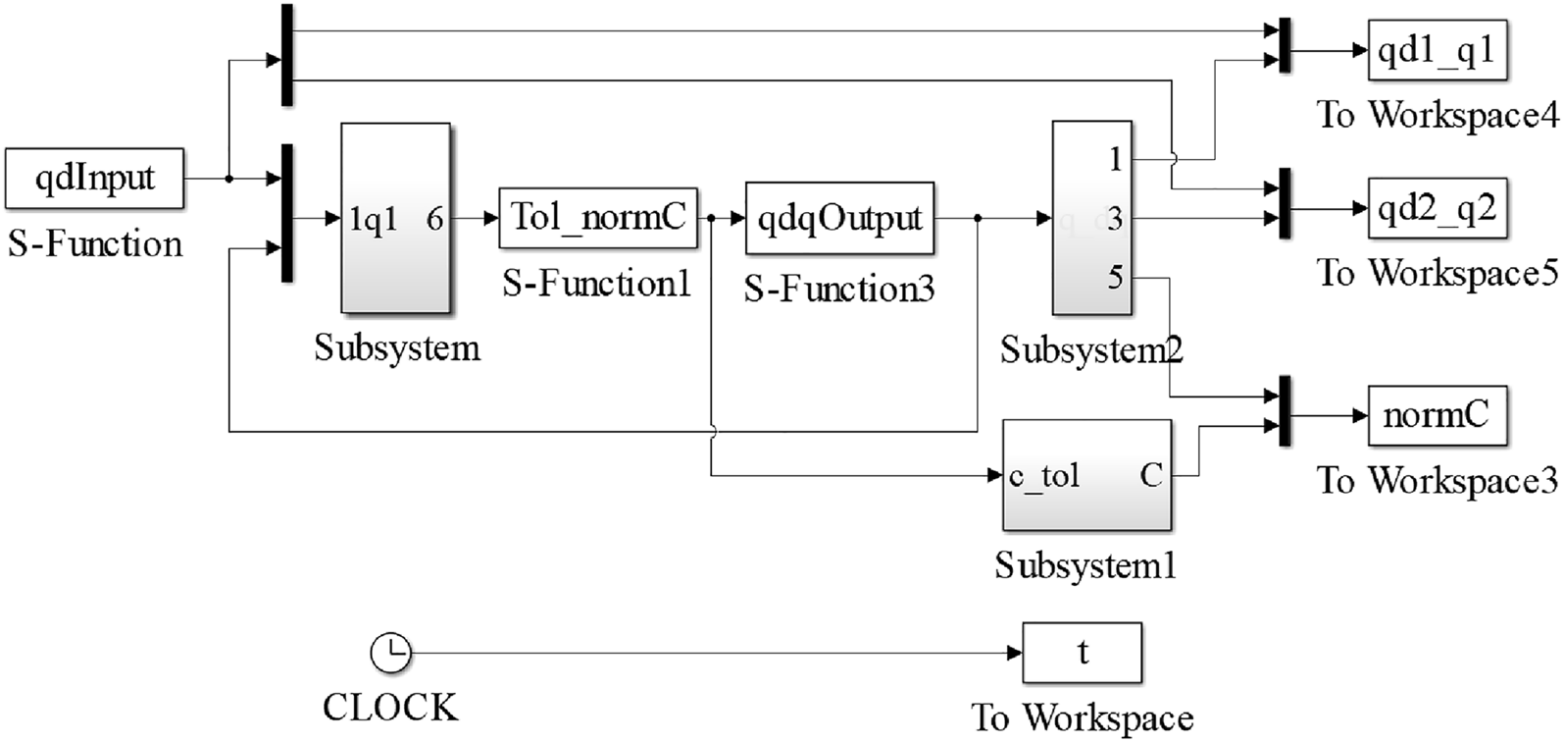
Model of robot joint control system.

The model of the robot joint control system is established by MATLAB/Simulink as shown in Fig. 1. The total simulation time is set to 40 s and the sampling frequency is 100 Hz.

From the joint sensor fault model and actuator fault model in (Eq. 5), they effect the system in a different way. However, through the model derivation and transformation, the sensor fault can be transformed into actuator fault through the first-order filter, which simplifies the analysis procedure. In order to reflect as much as information using collected fault data set, the sensor and actuator fault data are pre-processed according to the following formula.

6 $$Fault = a\Delta Sensor + bActuator + Noise$$

where $Fault$ denotes fault data set needed, $\Delta Sensor$ represents the difference between sensor output and settings, $Actuator$ is the output of actuator, $a$ and $b$ are sensor and actuator faults coefficient respectively. Item $Noise$ represents the gaussian white noise, and it adds with sensor and actuator feedback signals, and three of them form the fault signals to verify the noise immunity ability of the RNN.

In our study, fault types for both sensor and actuator are considered, including constant gain fault, constant deviation fault, stuck fault, and inactive fault. Above eight kinds of faults are researched separately for sensor and actuator. furthermore, constant deviation fault occurred simultaneous on sensor and actuator is also considered. Table 2 lists all ten kinds of system types including normal condition.

**Table 2:** Actuator fault type table.

Fault types	Parameters
ErrA1-F1	$\rho = 1,f_{a} = 5$
ErrA2-F2	$\rho = 0.8,f_{a} = 0$
ErrA3-F3	$\rho = 0,f_{a} = 30$
ErrA4-F4	$\rho = 0,f_{a} = 0$
Actuator & sensor deviation fault-F5	$$\left\{ \begin{gathered} \rho = 1,f_{a} = 5 \hfill \\ \lambda = 1,f_{b} = 0.1 \hfill \\ \end{gathered} \right.$$
ErrS1-F6	$\lambda = 1,f_{b} = 0.1$
ErrS2-F7	$\lambda = 0.8,f_{b} = 0$
ErrS3-F8	$\lambda = 0,f_{b} = 0.3$
ErrS4-F9	$\lambda = 0,f_{b} = 0$
Norm-F10	$$\left\{ \begin{gathered} \rho = 1,f_{a} = 0 \hfill \\ \lambda = 1,f_{b} = 0 \hfill \\ \end{gathered} \right.$$

## Fault diagnosis based on resnet

### Architecture of RESNET

RESNET is a kind of deep CNN, and it provides forward information through residual feedforward. Not only the depth of the network is expanded, but also the gradient disappearance phenomenon in the process of parameter updating is avoided by RESNET, which improves the strong adaptive capacity of the network.

**Figure 2:**
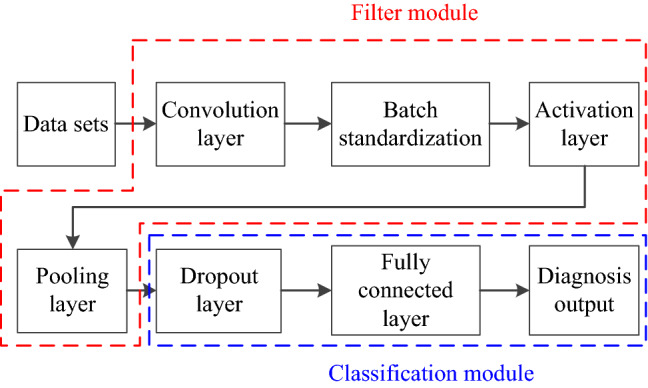
Diagram for CNN fault diagnosis system.

Generally, CNN consists of five parts: convolutional layer, batch standardization, active layer, pooling layer, and dropout layer. The whole CNN architecture is constructed by fully connected layer (FCL), as shown in Fig. 2.

Furthermore, CNN fault diagnosis architecture could be divided into two parts, and they are filtering and classification module. The filtering module can extract features from the input data set, and the classification module can process and classify the features extracted from the filtering module. Both the two modules use parameter sharing mechanism and sparse connection to reduce the amount of model training procedure and to improve the training efficiency of the network^36^.

### Enhancement of dataset

**Figure 3:**
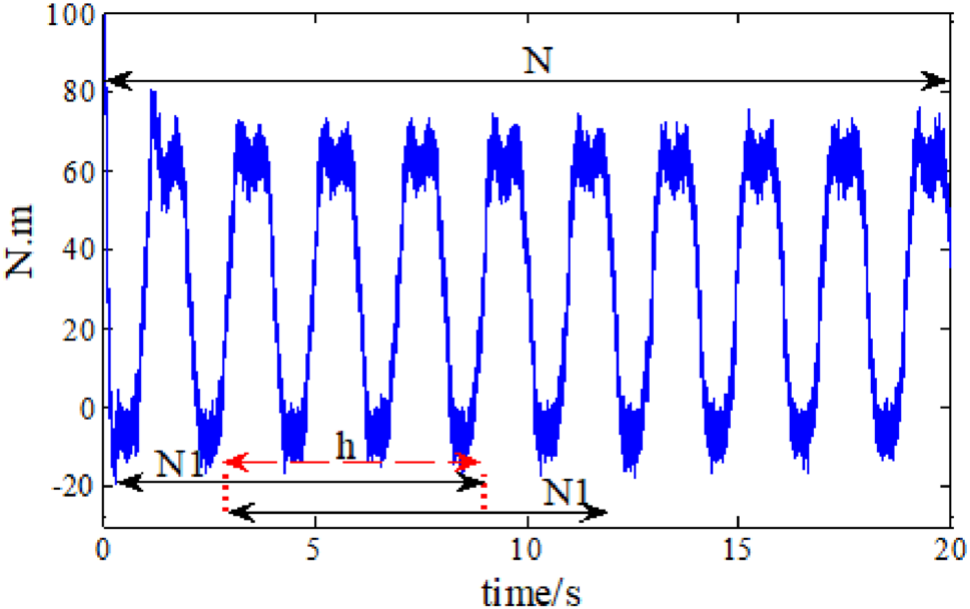
Schematic diagram of data set enhancement method.

It usually needs large training sample when using RESNET for fault diagnosis. Traditional data set enhancement methods mainly include: flip transformation, scaling, rotation transformation, mirror transformation, and translation transformation^37^. However, these methods are not suitable for one-dimensional sample data. In order to expand the acquired robot joint fault data, a sliding sampling data set enhancement method is proposed, as indicated in Fig. 3.

The system data over a period is obtained, and data sampling points of $N_{1}$ is used each time the network needs to be trained. Assuming a total number of $m*N_{1}$ data is obtained, then the network could be trained by $m$ times according to above method. In order to expand the coefficient of utilization for data, the start point of the second data set is $h$ backwards then the first one, and the rest is roughly the same.

When the sliding step size is small, that means $h$ is quite large, we can get more data samples, which can well meet the needs of data sets for deep convolution neural network in training. Comprehensive consideration, the sliding step selected here is 29.

### RESNET fault diagnosis model based on data driven

**Figure 4:**
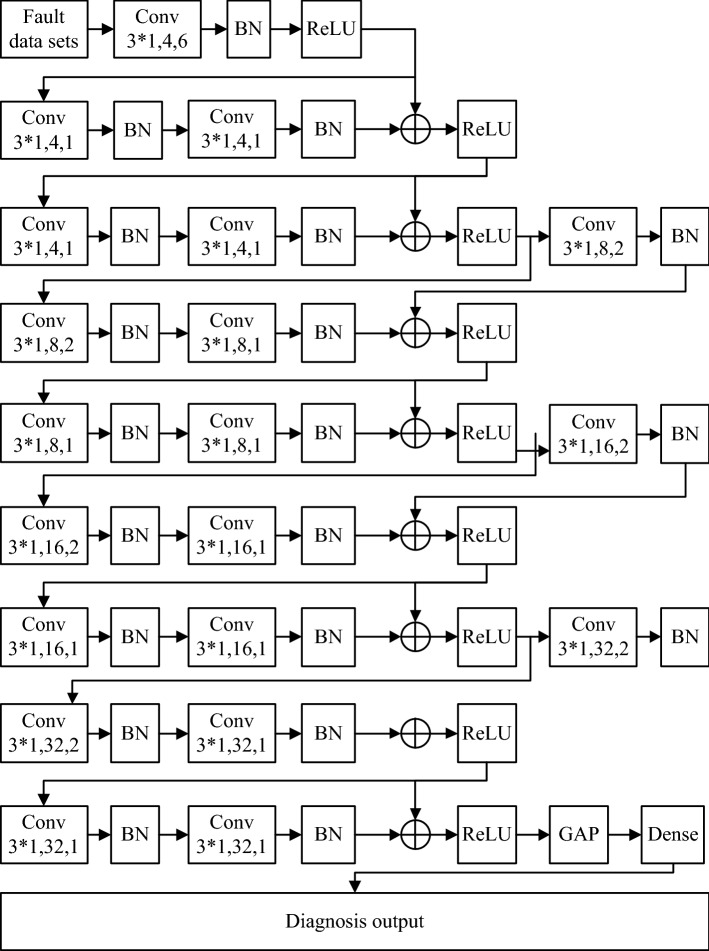
RESNET model diagram.

RESNET is a kind of deep convolutional neural network. In order to reduce the use of the model parameters and the calculation of the network, this paper uses several small convolution kernels and stacks them all, thus the convolution kernel size is increased, which enhances the ability of feature extraction for the network. The whole structure using RESNET in fault diagnosis from data input to fault diagnosis output is shown in Fig. 4.

Conv3*1,4,6 in Fig. 4 represents a convolution kernels dimension, and its depth is 4, sliding step is 6. BN, ReLU, GAP and Dense are batch standardization, active layer, pooling layer and fully connected layer respectively in Fig. 2. The structure of the whole network is regularized to prevent over fitting, when input and output dimensions of each convolution module are inconsistent, the residual jump is carried out through virtual channel, which is indicated by dotted line in Fig. 4.

## Simulation verification and analysis

The simulation platform used is PyCharm, combining with Python3.6 interpreter. RESNET architecture is based on Keras with Tensorflow as its background. Tensorflow is an end-to-end open-source machine learning platform, and it has a comprehensive and flexible ecosystem, which contains a variety of tools, libraries, and community resources. Keras is an open-source artificial neural network library written by python. It can be used as a high-level application interface of tensorflow to design, debug, evaluate, apply, and visualize the deep learning model^38^.

### Fault dataset

A variety of fault types listed in Table 1 are studied. The fault data sets are obtained for each kind of sensor and actuator faults, and we get 4000 data for every kind of fault type, around 216 data points are contained in the aforementioned 4000 data points. The sliding sampling enhancement method is used, as shown in Fig. 3, and 1000 samples are taken for each kind of fault, so a total of 10,000 samples are studied, including one data set under normal operation environment. The fault diagnosis results need to be evaluated, so 1000 samples are divided a by a ratio of 7:2:1, which means 70% of the data is used to train the neural network, 20% is used for validation and the rest for testing.

### Hyper-parameters of RESNET

According to previous research, hyper parameters in deep learning algorithm not only affect the performance of the algorithm itself, but also affect the expression ability of the trained model. However, about how to set hyper parameters, there is no mature theory. At present, most of the hyper parameter settings are based on the trial and error. This paper takes the advice from Y.bengio^39^. The corresponding hyper parameters are set according to whether these hyper parameters can increase or decrease the capacity of the model.

In the process of parameter updating, the exponential decay learning rate is used. At first, a large learning rate is used to get the optimal solution quickly, and then the learning rate is gradually reduced to keep the model stable in the later stage of training. The initial learning rate $\eta_{0}$ is set to 0.2, and decay learning rate $\xi$ is set to 0.99, so the decay rate is updated per round. The expression for decay rate is as follows.

$$\left\{ \begin{gathered} \eta = \eta_{0} \times \xi^{(H/L)} \hfill \\ H = Epoch/Batch\_k \hfill \\ \end{gathered} \right.,$$

where $\eta$ denotes exponential decay learning rate, $H$ stands for the current round, and $L$ is the period,$Batch\_k$ is the number of iterations. When a complete data set passes through the neural network once and then returns, this process is called $Epoch$ and here we set $Epoch$ to 40.

In order to alleviate the over fitting of neural network, $l_{2}$ regularization method is used in this paper. Regularization is to introduce the model complexity index into the loss function and suppress the noise in the training data set by weighting the parameters $w$ in the neural network. The loss function is as follows.

7 $$\left\{ \begin{gathered} Loss = Loss\_all + REGULARIZER \times Loss(w) \hfill \\ Loss(w) = \sum\limits_{i} {\left| {w_{i} } \right|} \hfill \\ \end{gathered} \right.,$$

where $Loss\_all$ represents loss function of all parameters in the model, $REGULARIZER$ is the regularization weight, $w$ generally refers to the weight in the forward propagation of neural network, and $Loss(w)$ is the result of $l_{2}$ regularization of parameter $w$.

The Adma optimal algorithm is used and the procedure of weight updating is as follows.

*Step 1*: Give the iteration step $\varepsilon = 0.001$.

*Step 2*: Set the decay rate for matrix calculation,$\rho_{1} = 0.99,\rho_{2} = 0.999$.

*Step 3*: Determine the convergence threshold $\delta = 10^{ - 8}$.

*Step 4*: Initialize network weight $\theta$,and initializes first and second moment variables $s,r$,and set $s = 0,r = 0$.

*Step 5*: Set the simulation time step to 0.0001.

*Step 6*: Small data set with $m$ samples are collected from the training set, use $\left\{ {x^{{(1)}} ,x^{{(2)}} ,...,x^{{\text{(m)}}} } \right\}$ denotes it and set corresponding goals $y^{(i)}$.

*Step 7*: Calculate gradient variable $g \leftarrow \frac{1}{m}\nabla_{\theta } \sum\limits_{i} {L{(}f{(}x^{{{(}i{)}}} {;}\theta {),}y^{(i)} {)}}$, and update biased first moment estimation $s \leftarrow \rho_{1} s + (1 - \rho_{1} )g$ as well as biased second moment estimation $r \leftarrow \rho_{2} r + (1 - \rho_{2} )g$.

*Step 8*: Correct the deviation of the first moment $\hat{s} \leftarrow \frac{s}{{1 - \rho_{1}^{t} }}$ and deviation of the second moment $\hat{r} \leftarrow \frac{r}{{1 - \rho_{2}^{t} }}$.

*Step 9*: Calculate incremental weight error $\Delta \theta = - \varepsilon \frac{{\hat{s}}}{{\sqrt {\hat{r}} + \delta }}$,and update it to the network weight $\theta \leftarrow \theta + \Delta \theta$.

*Step 10*: If the convergence threshold in Step3 is not met, then, back to Step6, else end the iterative process.

Machine learning is a highly empirical process, accompanied by a large number of iterations. In order to find the most suitable model, a lot of model training is needed. The optimizer is a tool to guide the neural network to update parameters. Using the optimization algorithm can help to quickly train the model and find the optimal solution. In this paper, we choose the Adma optimization algorithm, which does not occupy memory, and it is an efficient random optimization algorithm.

### Fault diagnosis results

In order to verify the feasibility and effectiveness of the RESNET fault diagnosis method used in this paper for robot joint sensor and actuator fault diagnosis, the artificial neural network, support vector machine, convolution neural network, long-term memory network and deep residual network methods are used for comparative analysis and verification. The average accuracy and standard deviation of different network training results are compared in Table 3.

**Table 3:** Actuator fault type table.

Network type	Average accuracy on training set	Average accuracy on testing set
ANN	$68.32 \pm 0.63$	$68.16 \pm 1.24$
SVM	$63.16 \pm 0.23$	$63.02 \pm 1.36$
CNN	$97.68 \pm 1.65$	$95.73 \pm 1.82$
LTMN	$99.87 \pm 0.12$	$98.67 \pm 0.24$
RESNET	$99.91 \pm 0.09$	$99.87 \pm 0.13$

It can be seen from Table 3 that compared with the traditional data-driven fault diagnosis method (ANN and SVM), the fault recognition accuracy of the deep learning fault diagnosis method is significantly improved. The average accuracy of network training results is less than 70% using traditional fault diagnosis method, but the average accuracy of CNN, LTMN and RESNET is more than 95%. Meanwhile, the average accuracy of RESNET in both training and test set is more than 99%, which means RESNET is suitable and effective for fault diagnosis of robot joint sensors and actuators faults.

**Figure 5:**
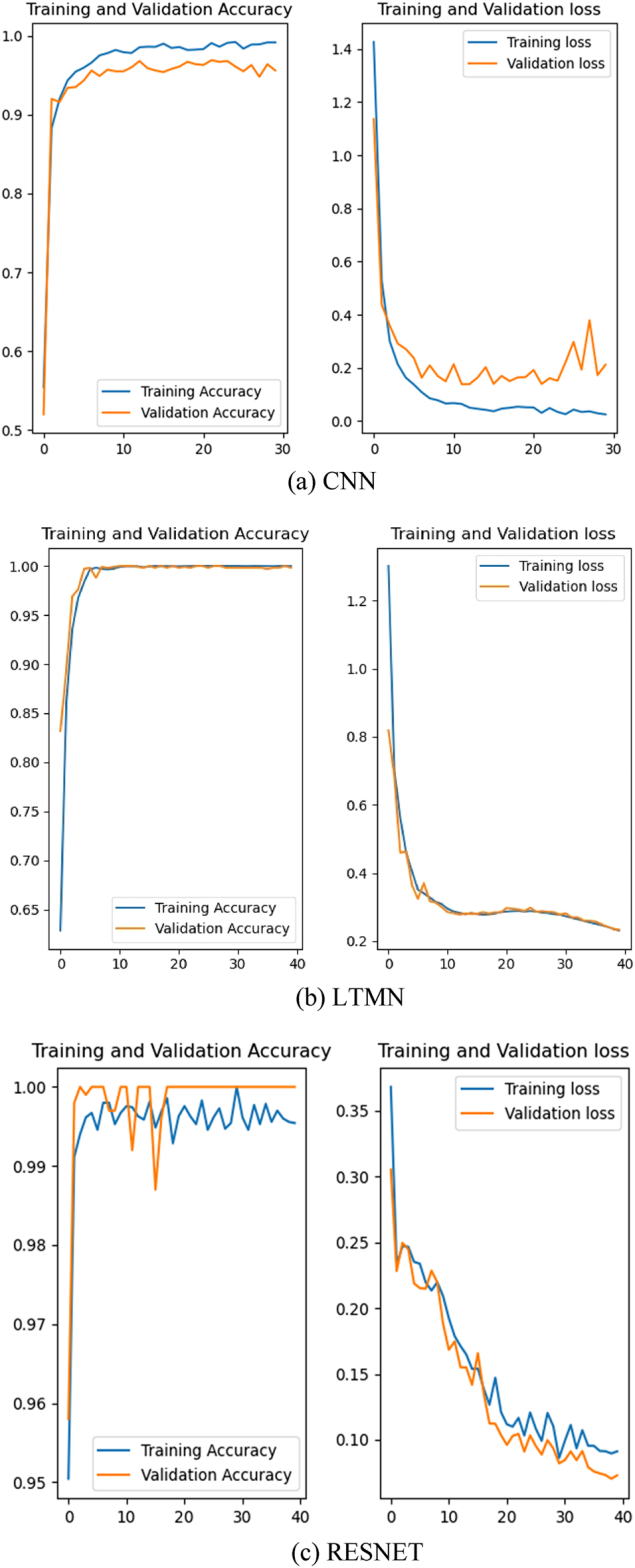
Fault diagnosis results of three kind of neural networks.

For the purpose of further analyzing the diagnosis effect of three kinds of deep neural networks (CNN, LTMN and RESNET) on robot joint sensor and actuator faults, the accuracy and loss function of each kind of network in the training set and testing set are drawn with the help of tensorflow, and the results are shown in Fig. 5 and Table 4.

**Table 4:** Actuator fault type table.

Network type	Max accuracy for training (%)	Max accuracy for testing (%)	Stable time	Training time
CNN	99.2	97.3	4 Epoch	1 T
LTMN	100	100	7 Epoch	12 T
RESNET	100	100	5 Epoch	2.5 T

Figure 5 shows the fault diagnosis accuracy and loss on both training and validation datasets. The reasons that validation loss is better than training loss may be deduced as follows. The datasets under the same working condition are grouped proportionally as 7:2:1, so the validation datasets only occupy 20% of all. This may cause the accuracy difference.

The dropout layer in training process. In order to accelerate the model training time, the dropout layer is introduced. While the dropout doesn’t operate at validation stage. Combining Fig. 5, Table 4, it can be seen that the highest accuracy of CNN in training data set is 99.2%, and that number is 97.3% in test data set. The highest accuracy of LTMN and RESNET in training data set and test data set is 100%, and their accuracy curve is relatively smoother. Obviously, both LTMN and RESNET can better reflect the fault information from the original data set and can make more accurate judgment of sensor and actuator faults in robot joints. But from the point of view of the training time, RESNET needs 2.5 T, while LTMN needs 12 T, which is more than five times of RESNET.

Thus, compared with other data-driven fault diagnosis methods, the deep RESNET network used in this paper has higher accuracy in the fault diagnosis of robot joint sensors and actuators, and the training time cost is relatively less, so it is more practical.

**Figure 6:**
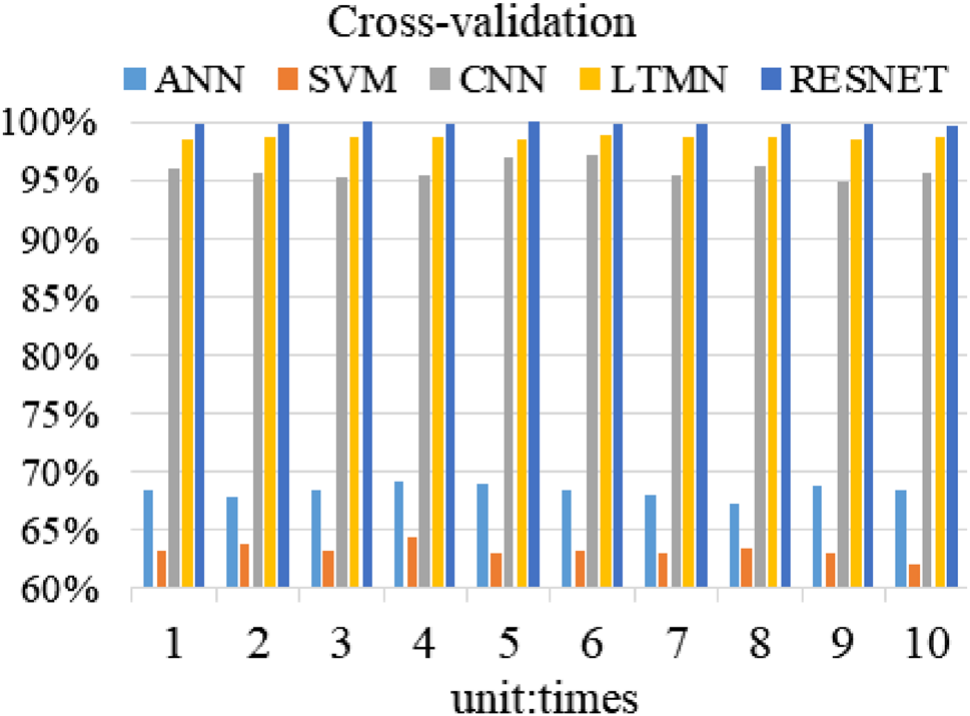
Cross validation result.

Considering that the initial value of the neural network is random, in order to avoid the inaccuracy of fault diagnosis results and verify the reliability of each training result, the cross method is used to train the fault data set for ten times. The results are shown in Fig. 6.

As can be seen from Fig. 6, the training accuracy of RESNET in each experiment is more than 99%, and the lowest accuracy is 99.6%. Therefore, it can be concluded that the best fault diagnosis method of robot joint sensor and actuator is RESNET-based neural network in our study.

### Visualization analysis of RESNET classification results

**Figure 7:**
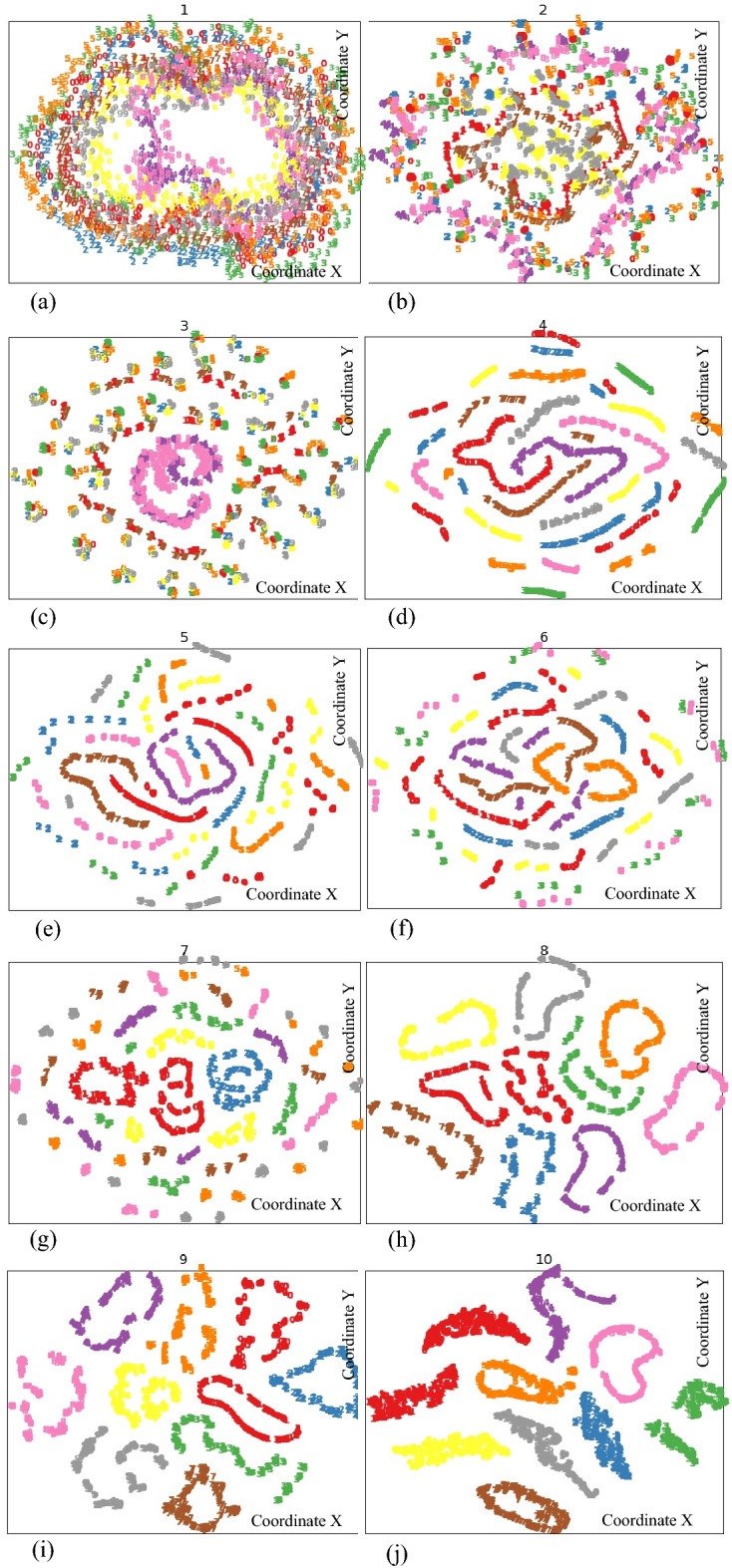
Output of of residual block.

Visualization analysis of RESNET is carried out to study the intermediate process of neural network. t-distributed stochastic neighbour embedding (t-SNE) is used to reduce the data dimension of the output of each residual block in RESNET architecture, and the results are shown in Fig. 7.

Figure 7a is the result of t-SNE visualization of fault data set with signal to noise ratio setting to 10. Figure 7b–i correspond to the t-SNE visualization results of the outputs of the eight residual blocks in the RESNET architecture in Fig. 4. Figure 7j is the final output of RESNET neural network. Different curves with different colours in Fig. 7 represent different fault types listed in Table 1. A total of 10 colours correspond to 9 faults and 1 health state for robot joint system.

It can be seen from the Fig. 7 that with the increase of residual blocks, the data expression ability of RESNET network is gradually enhanced, and finally the accurate classification of nine kinds of fault types is realized. From the visualization analysis results of data in two-dimensional space, the feasibility and effectiveness of RESNET fault diagnosis algorithm proposed in this paper are further verified.

## Conclusion

A novel RESNET based neural network fault diagnosis method for robot joint system is proposed in this paper. Aiming at the problem that the traditional robot joint fault diagnosis algorithm cannot accurately locate the fault, a data-driven RESNET fault diagnosis algorithm is proposed. The fault models of robot joint sensor and actuator are built to simulate various fault states in order for fault data acquisition. After getting the fault data set needed, the RESNET architecture constructed by small convolution kernel is studied, and by increasing the residual blocks, the convolution kernel is gradually increased to improve the fault extraction ability of the model. Compared with other data-driven based fault diagnosis algorithms, simulation results show that the accuracy of fault diagnosis based on RESNET is more than 99%, which is the highest among all studied methods and meanwhile the model training time is less.

## Data Availability

The data presented in this study are available on request from the corresponding author.

## Code availability

The data presented in this study are available on request from the corresponding author.
